# The scientific evidence for a potential link between confusion and urinary tract infection in the elderly is still confusing - a systematic literature review

**DOI:** 10.1186/s12877-019-1049-7

**Published:** 2019-02-04

**Authors:** Sean Mayne, Alexander Bowden, Pär-Daniel Sundvall, Ronny Gunnarsson

**Affiliations:** 1 Cairns Clinical School, College of Medicine and Dentistry, James Cook University, PO Box 902, Cairns, Queensland 4870 Australia; 2 Cairns Hospital, Queensland Health, Cairns, Queensland Australia; 3 Research and Development Unit, Primary Health Care in Southern Älvsborg County, Sven Eriksonsplatsen 4, SE-503 38 Borås, Sweden; 4 Department of Public Health and Community Medicine, Institute of Medicine, Sahlgrenska Academy at the University of Gothenburg, Gothenburg, Sweden

**Keywords:** Confusion, Delirium, Urinary tract infection, Bacteriuria, Elderly

## Abstract

**Background:**

Non-specific symptoms, such as confusion, are often suspected to be caused by urinary tract infection (UTI) and continues to be the most common reason for suspecting a UTI despite many other potential causes. This leads to significant overdiagnosis of UTI, inappropriate antibiotic use and potential harmful outcomes. This problem is particularly prevalent in nursing home settings.

**Methods:**

A systematic review of the literature was conducted assessing the association between confusion and UTI in the elderly. PubMed, Scopus and PsychInfo were searched with the following terms: confusion, delirium, altered mental status, acute confusional state, urinary tract infection, urine infection, urinary infection and bacteriuria. Inclusion criteria and methods were specified in advance and documented in the protocol, which was published with PROSPERO (registration ID: CRD42015025804). Quality assessment was conducted independently by two authors. Data were extracted using a standardised extraction tool and a qualitative synthesis of evidence was made.

**Results:**

One thousand seven hunderd two original records were identified, of which 22 were included in the final analysis. The quality of these included studies varied, with frequent poor case definitions for UTI or confusion contributing to large variation in results and limiting their validity. Eight studies defined confusion using valid criteria; however, no studies defined UTI in accordance with established criteria. As no study used an acceptable definition of confusion and UTI, an association could not be reliably established. Only one study had acceptable definitions of confusion and bacteriuria, reporting an association with the relative risk being 1.4 (95% CI 1.0–1.7, *p* = 0.034).

**Conclusions:**

Current evidence appears insufficient to accurately determine if UTI and confusion are associated, with estimates varying widely. This was often attributable to poor case definitions for UTI or confusion, or inadequate control of confounding factors. Future well-designed studies, using validated criteria for UTI and confusion are required to examine the relationship between UTI and acute confusion in the elderly. The optimal solution to clarify this clinical issue would be a randomized controlled trial comparing the effect of antibiotics versus placebo in patients with new onset or worsening confusion and presence of bacteriuria while lacking specific urinary tract symptoms.

## Background

It is well documented that lower urinary tract infection (UTI) should only be diagnosed when there are new onset localising genitourinary signs and symptoms and a positive urine culture result [1]. However, despite new onset or worsening of confusion being a non-specific symptom, it continues to be the most common reason for suspecting a lower UTI in elderly patients, often leading to antibiotic treatment [2–4]. The diagnosis of UTI is further complicated by the high prevalence of asymptomatic bacteriuria, particularly in nursing home residents, varying between 25 and 50% for women and 15–40% for men, without an indwelling urinary catheter [5]. While urine cultures can guide the choice of antibiotic, their poor positive predictive value means a positive culture alone should not warrant initiation of antibiotics [6]. Additionally, evidence suggests patients with confusion and dementia are more likely to be continuously bacteriuric [7]. Due to all of these confounding factors, new onset or worsening of non-specific symptoms in residents is one of the major diagnostic challenges in caring for the elderly.

Subsequently, many different consensus based criteria to enable appropriate diagnosis of UTI have been devised, most notably the revised Mcgeer and updated Loeb criteria [1, 8]. Despite the difficulty of diagnosing UTI, there is sound evidence that elderly residents with symptomatic lower UTI should receive antibiotic treatment and elderly residents with asymptomatic bacteriuria should not [9, 10]. Although inappropriate antibiotic use results in those few residents suffering from a lower UTI to be treated promptly, it leads to significant overdiagnosis of UTI and potentially harmful outcomes through misdiagnosis. With many residents receiving unnecessary antibiotics with possible adverse reactions, and the ever-increasing rates of antibiotic resistance, it is evident that inappropriate antibiotic use in this population must be reduced [11].

A previous literature review conducted by Balogun et al. exclusively reviewed the association between symptomatic UTI and delirium in the elderly in five publications. It concluded that there may be an association between symptomatic UTI and delirium; however, some methodological flaws may have led to biased results [12]. It was limited by only using the term delirium and excluding terms like confusion, resulting in many publications potentially being excluded. In addition, Balogun et al. only searched the Medline database potentially missing important publications.

UTI is a broad diagnosis encompassing infections arising from all levels of the urinary tract, ranging in severity from acute cystitis to acute pyelonephritis. This systematic review aims to review the evidence for the association between acute cystitis or bacteriuria and confusion in the elderly in all care settings.

## Methods

### Protocol and registration

The review was conducted in accordance with the Preferred Reporting Items for Systematic Reviews and Meta-Analyses (PRISMA) statement [13]. Inclusion criteria and methods were specified in advance and documented in the review protocol. The initial protocol was published with PROSPERO International prospective register of systematic reviews, registration number CRD42015025804.

### Eligibility criteria

This review included quantitative studies which met the following inclusion criteria:

- Elderly participants. The majority of the participants in the study must be representative of an elderly population, defined as the median or mean age ≥ 65 years.
- Primary studies in which participants with lower UTI or bacteriuria were assessed for concurrent confusion, or participants with confusion were assessed for concurrent UTI or bacteriuria.
- Any care setting (Hospital, Community, Long Term Care Facility).

The following exclusion criteria were applied:

- Studies that refer to specific sub-populations of patients with UTI. Examples include: stroke patients, Alzheimers or dementia patients or post-surgical patients
- Studies that exclusively report catheter associated UTI;
- Non-English publications
- Case studies

The primary outcomes of interest were confusion, UTI and bacteriuria. Due to the large variety of terminology that surrounds the symptom of confusion, definitions used for confusion in this review encompassed: confusion, acute confusional state, delirium, altered mental status, altered mental state. This broad definition was used so as to capture all studies which may have assessed confusion but used alternative terminology. No absolute lower age limit was set, as it was anticipated that these would have an overall negligible effect on the data. This was so as to not exclude studies which may present data representative of an elderly population but which included a small minority of non-elderly participants. Studies that referred to specific subpopulations, or exclusively reported catheter associated UTI were also excluded so as to explore the association between confusion and UTI or bacteriuria in a general elderly population.

### Identification of studies

Three databases were accessed to identify studies eligible for this review: PubMed, Scopus and PsycINFO (via ProQuest). The search terms were related to three key topics: confusion, UTI and bacteriuria (Table 1) with adaptations for Scopus and PsycINFO. No restrictions on date or language were applied and studies published up to August 2015 were included. Once the final list of full text articles was determined, the references and citation history of the included studies were screened for other potential studies eligible for the review. The full texts of any new studies deemed possibly eligible for inclusion, were then retrieved and assessed.

**Table 1:** PubMed Search Strategy

“Delirium”[Mesh] OR “Confusion”[Mesh] OR “acute confusional state”[All Fields] OR “altered mental status”[All Fields] OR “altered mental state”[All Fields] OR “delirium”[All Fields] OR “confusion”[All Fields]	
AND	
“Urinary Tract Infections”[Mesh] OR “Bacteriuria”[Mesh] OR “urinary infection”[All Fields] OR “urine infection”[All Fields] OR “urinary tract infection”[All Fields] OR “Bacteriuria”[All Fields]	

### Study selection

After each database had been searched, the search results were collated and duplicates removed. Titles and, where available, abstracts retrieved were assessed for eligibility against the described inclusion criteria. Studies that clearly did not meet the review’s criteria were excluded. The full texts of potentially eligible studies and those that after title and abstract screening were not able to be definitively excluded were retrieved. The full text articles were then assessed for eligibility by the first reviewer (SM). Studies that could not be definitively excluded were discussed and resolved by consensus with another reviewer (RG). Studies excluded at this stage were recorded and their reason for exclusion is reported.

### Data extraction

Data extraction was performed by one author (SM) using a standardised, pre-designed data extraction form, with the exception of data relevant to the quality assessment which was extracted by two review authors (SM and AB) independently. Any discrepancies identified were resolved by consensus, or through discussion with the third reviewer (RG). Data extracted from each eligible study included:

- General information: author, year of publication, title, type of publication, journal;
- Study characteristics: aim, study design;
- Patient sample: number, age, gender, presence of urinary catheters;
- Care setting: Hospital, Long-term Care Facility, Community;
- Confusion criteria: criteria utilised to diagnose confusion;
- UTI/Bacteriuria criteria: criteria utilised to diagnose UTI/bacteriuria;
- Results: association between UTI/bacteriuria and confusion if reported, rates of patients with UTI/bacteriuria having confusion, rates of patients with confusion having UTI/bacteriuria;
- Risk of bias: see Quality Assessment below

### Quality assessment / risk of Bias

Two review authors (SM and AB) assessed the risk of bias of included studies independently, with any discrepancies being resolved by consensus, or through discussion with a third reviewer (RG), if necessary. The risk of bias was assessed using a modified version of the assessment checklist developed by Downs and Black [14]. Quality items that pertained to interventions and trial studies were removed as they were not deemed to be appropriate for the studies included in this review. An additional five quality items were added to the quality assessment to determine if studies described the criteria used for confusion, UTI and bacteriuria, and if their criteria for UTI and confusion were valid and reliable. Criteria for confusion were deemed valid and reliable if accepted criteria were utilised, including: the Confusion Assessment Method, the Organic Brain Syndrome Scale or the Diagnosis and Statistical Manual (DSM) criteria [15–17]. Similarly, criteria for UTI were deemed valid and reliable if established criteria for UTI were utilised, including: the McGeer Criteria, the revised McGeer Criteria, the Loeb Criteria, or the Revised Loeb Criteria [1, 8, 18, 19]. The modified checklist finally consisted of 14 quality items, grouped into: reporting, internal validity, external validity and criteria (Table 2). The risk of bias for each quality item was reported as low risk of bias, high risk of bias, unclear risk of bias or not applicable.

**Table 2:** Quality Assessment Criteria

Item Number	Category	Quality Assessment
1	Reporting	The main outcomes of the study to be measured are clearly described in the Introduction or Methods section
2	Reporting	The characteristics of the patients included in the study are clearly described (ie. Inclusion and Exclusion Criteria stated, case definition and the source for controls stated in case control studies)
3	Reporting	The number/characteristics of non-responders (cross-sectional) or patients lost to follow-up (longitudinal) have been described
4	Reporting	The study provides estimates of the random variability in the data for the association of UTI or Bacteriuria and confusion
5	Reporting	Actual probability values have been reported for the association between UTI and Delirium eg. *p* = .035 not *p* < 0.5, except where *p* < 0.001
6	Internal Validity	The statistical tests used to assess the association of UTI or Bacteriuria and confusion were appropriate.
7	Internal Validity	The distribution of principle confounders in each comparison group were clearly described
8	External Validity	Patients asked to participate in the study were representative of the entire population of which they were recruited (source population identified and those asked to participate were either the entire population or a randomised sample of the entire population)
9	External Validity	Those participants who were prepared to participate, were representative of the entire population of which they were recruited? > 70% = Yes, < 70% = No
10	Criteria	The criteria used to define caseness for UTI was described
11	Criteria	The criteria used to define caseness for UTI was valid and reliable
12	Criteria	Criteria for Bacteriuria was described
13	Criteria	The criteria used to define caseness for confusion was described
14	Criteria	The criteria used to define caseness for confusion was valid and reliable

### Data synthesis

Although the broad search strategy described was employed to enable the meta-analysis of data from included studies if deemed feasible, due to the heterogeneity of the data and the variety of definitions for confusion and UTI reported between the studies, meta-analysis was not conducted. Alternatively, a qualitative synthesis of the findings from the included studies was performed, structured around the quality of data, consistency of definitions and the evidence for the association between confusion and UTI.

## Results

### Study selection

**Fig. 1:**
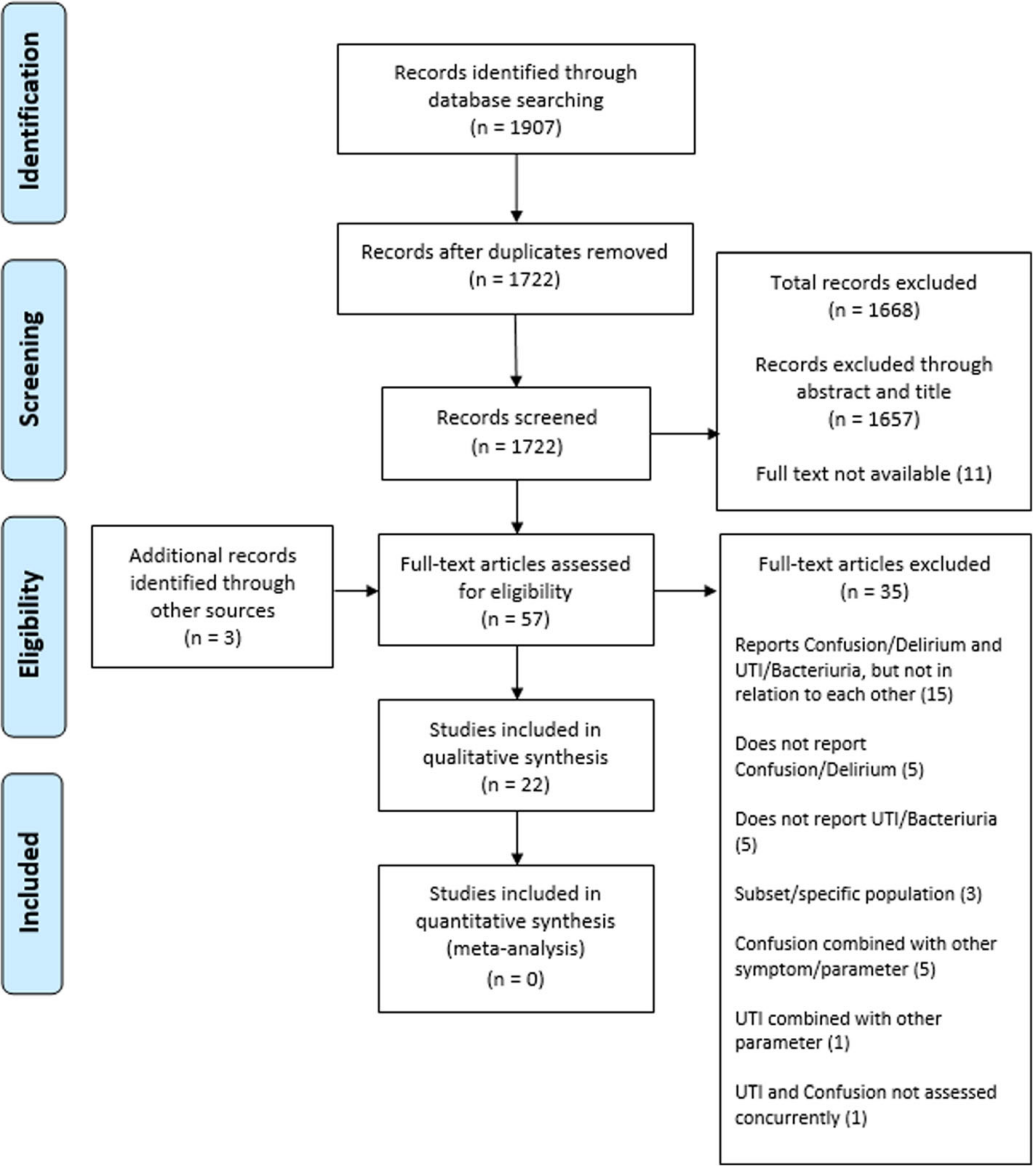
Flow diagram showing identification of studies for inclusion in this systematic review according to PRISMA guidelines

Searches identified a total of 1907 records (Fig. 1). After duplicate records were removed, 1722 remained. These articles were then screened by title and abstract against the inclusion and exclusion criteria, leading to the exclusion of 1657 articles, as it appeared they clearly did not fulfil the eligibility criteria. Eleven potential records were excluded as their full texts were unable to be obtained. The full texts of the remaining 54 articles were closely examined. Thirty-five articles were excluded at this stage, with the most common reason being the study reported confusion and UTI/bacteriuria in their population, but not in relation to each other [20–34]. Other common reasons included: the study did not report confusion [35–39], the study did not report UTI/bacteriuria [2, 40–43], the study only reported the association in a specific subpopulation [44–46], or the study combined their measurement of confusion with other parameters [7, 47–50]. Two studies were also excluded as UTI and confusion were not assessed concurrently [51] and reported UTI was combined with other parameters [52]. Three additional studies that met the inclusion criteria were identified by searching the references of relevant articles and searching for studies that cited these articles. No studies were deemed suitable for quantitative synthesis. A total of 22 articles met the inclusion criteria and were included in the systematic review.

### Quality assessment

**Fig. 2:**
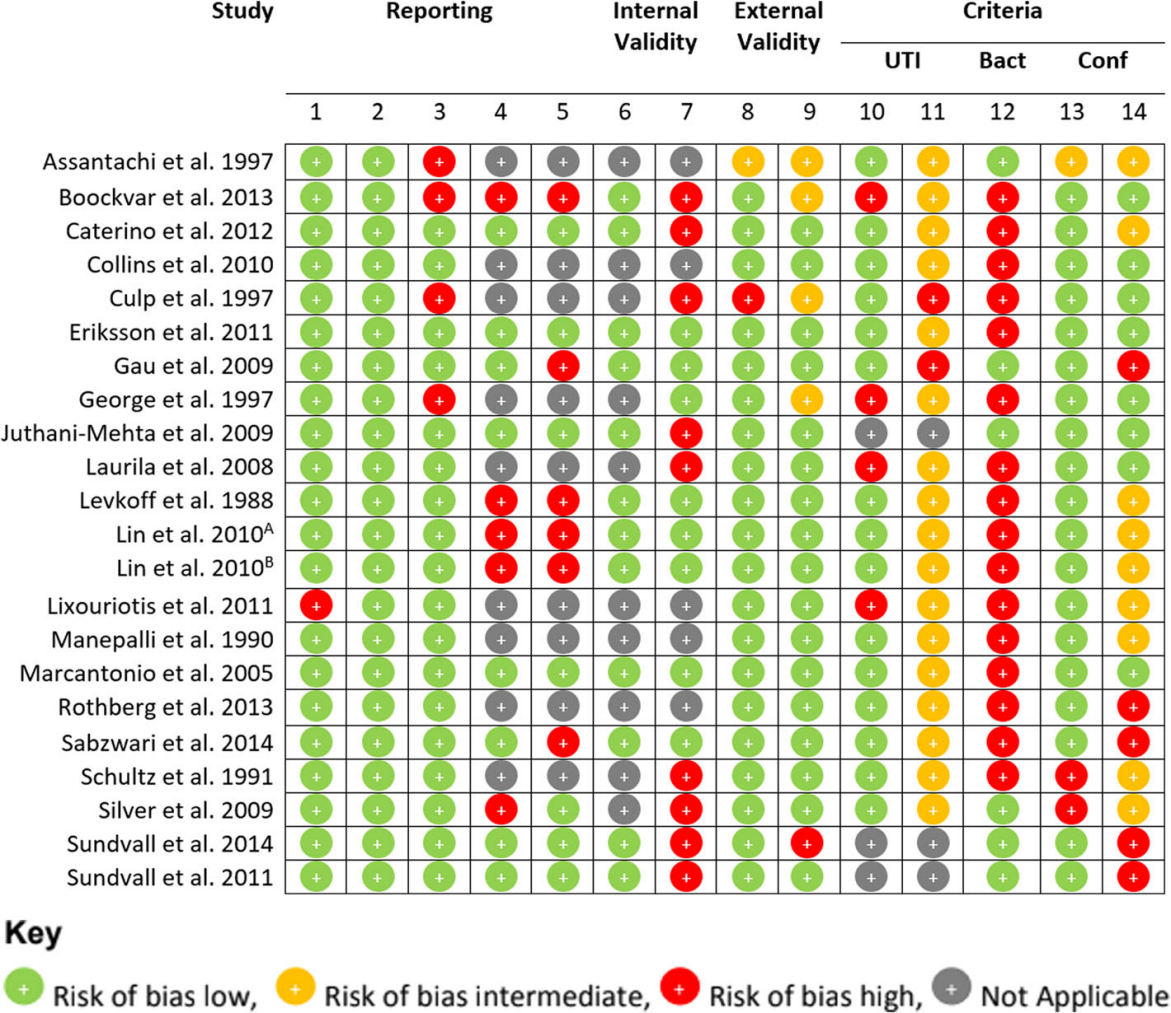
Quality Assessment

The quality of the studies included in this review varied considerably (Fig. 2). This is partially due to inclusion of all studies that reported data on the rate of confusion in patients with suspected UTI or bacteriuria, or vice versa, even if it was not the main objective of the study. Reporting of main outcomes, description of patient characteristics and number of non-responders/patients lost to follow up were done well in most studies, with only one small study not clearly describing their main outcomes [53] and four studies not reporting the number of non-responders or patients lost to follow up [40, 54–56]. In terms of internal validity, all applicable studies were deemed to have used appropriate statistical tests; however, half of the studies did not clearly describe other principle confounders in their comparison groups. The external validity, however, of all studies, was generally very high.

The quality of the criteria used to define UTI, bacteriuria and confusion varied considerably and was generally quite poor. No studies used criteria for UTI completely consistent with the revised Mcgeer or Loeb Criteria. Two studies employed a reproducible definition of UTI although neither employed published explicit criteria developed through expert consensus. [57, 58]. Many studies utilised discharge coding from patient databases which resulted in the reliability of their criteria being unable to be determined [59–65]. Two studies were identified that used criteria that were clearly inappropriate [66, 67]. Three studies did not provide a definition for UTI, as they reported confusion in association with validated criteria for bacteriuria only [3, 68, 69]. Only one of these studies utilised an appropriate definition of bacteriuria and validated criteria for delirium [3]. Three of the studies which provided a definition for UTI also defined criteria for bacteriuria [56, 58, 67].

Almost all studies provided a definition of confusion criteria, but only eight studies used criteria that were valid and reliable (Table 3) [3, 54, 55, 60, 66, 70–72]. Five studies used criteria for confusion which were clearly not valid or reliable [65, 67–69, 73], and nine were unclear in their criteria used (Table 4) [53, 56–59, 61–64].

**Table 3:** Summary of Studies using Valid Criteria for Confusion

*Study*	*Design of Study*	*Patient Sample Number, Age, Female %, Catheter %*	*Care Setting*	*Association between Confusion and UTI Primary Aim of Study*	*Confusion Diagnostic Criteria*	*UTI/Bacteriuria Diagnostic Criteria*	*Results*
*Boockvar* et al. *2013* [54]	Cohort; Prospective	136 patients Age: mean 76 years, SD 12 Female 40% Catheter: Unclear	Nursing Home	Partial	Delirium CAM	Bacteriuria Not Stated UTI Not described	11 out of 43 (26%) incidents of UTI had delirium
*Collins* et al. *2010* [60]	Cross Sectional; Prospective	710 patients Age: ≥ 70 years; mean 83, range 70–101 Female: 60% Catheter: 5.5%	Hospital (Medical Acute Admissions Unit)	Partially	Delirium CAM	Bacteriuria Not Stated UTI ICD – 10 Codes	16 out of 110 (15%) patients admitted with delirium had UTI
*Culp* et al. *1997* [66]	Cross Sectional; Prospective	37 residents Age: ≥65 years; Female: Unclear Catheter: Unclear	Long term care facilities (intermediate and skilled bed)	Partially	Delirium screened using NEECHAM and confirmed with CAM	Bacteriuria Not Stated UTI Defined by leukocyturia	7 out of 15 (47%) patients with delirium had UTI
*Eriksson* et al. *2011* [70]	Cross Sectional; Prospective	504 citizens from population record, Age: 172 aged 85 years, 169 aged 90 years, 63 aged ≥95 years, Female: 100% Catheter: 1.8%	Community Setting (Institutionalised care: 238/504)	Yes	Delirium Organic Brain Syndrome Scale	Bacteriuria Not Stated UTI documented symptomatic UTI, with short- or long-term antibiotics, or symptoms and laboratory tests judged to indicate a UTI	39 out of 87 (45%) patients with UTI had delirium 39 out of 137 (29%) patients with delirium had UTI UTI was present in 29% of patients with delirium and 13% of those without delirium (p < 0.001) UTI was associated with delirium OR 1.9 (95% CI 1.1–3.3, *p* = 0.025)
*George* et al. *1997* [55]	Case Controlled Prospective	171 delirious patients Age: ≥65 years mean 81, range 65–98 Female: 54% Catheter: Unclear	Hospital	Partial	Delirium DSM III Criteria	Bacteriuria Not Stated UTI Not Stated	25 out of 171 (15%) patients with delirium had UTI
*Juthani-Mehta* et al. *2009* [3]	Cohort Study Prospective	551 Residents Age: ≥65 years mean 86, SD 7.1 Female:81% Catheter: 0%	Long Term Care Facilities	Partial	Change in Mental Status Adapted delirium criteria from DSM VI	Bacteriuria ≥10^4^ cfu/ml on urine culture + pyuria (> 10 WBC) on urinalysis UTI Not Stated	70 out of 147 (48%) patients with bacteriuria + pyuria had mental status changes 70 out of 170 (41%) patients with mental status change had bacteriuria + pyuria Association of mental status change with bacteriuria +pyuria RR 1.4 (95% CI 1.0–1.7, p = 0.034)
*Laurila* et al. *2008* [71]	Cohort Study Prospective	87 patients Age: ≥70 years mean 83.8, range 71–97 Female: 76% Catheter: Unclear	Hospital (Medical Ward)	Partial	Delirium DSM-IV	Bacteriuria Not Stated UTI Consensus of three geriatricians after assessment	35 of 87 (40%) patients with delirium had UTI
*Marcantonio* et al. *2005* [72]	Cohort; Prospective	188 patients with delirium Age: ≥ 65 years mean 83.3, SD 7.4 Female: 68% Catheter: Unclear	Hospital (Post-acute admissions)	No	Delirium CAM	Bacteriuria Not Stated UTI Clinically documented in the medical record	22 out of 188 (12%) patients with delirium had UTI 12% of patients with delirium had UTI compared to 7% of patients without delirium (*p* = 0.22)

**Table 4:** Summary of Studies with Invalid/Biased Criteria for Confusion

*Study*	*Design of Study*	*Patient Sample Number, Age,* *Female %, Catheter %*	*Care Setting*	*Association between Confusion and UTI Primary Aim of Study*	*Confusion Diagnostic Criteria*	*UTI/Bacteriuria Diagnostic Criteria*	*Results*
*Assantachai* et al. *1997* [56]	Cross Sectional; Prospective	100 patients with UTI, Age: ≥ 60 years, mean 72 +/− 8.6, range 60–100 Female: 78% Catheter: 46%	Hospital (General and Intensive care wards 95:5)	No	Confusion Not stated	Bacteriuria ≥10^5^ bacteria/ml UTI Not described	60 out of 100 (60%) patients with UTI had Confusion
*Caterino* et al. *2012* [59]	Cross Sectional; Retrospective	25.4 million presentations of UTI Age: ≥ 18 years, 18,200,000 aged 18–64, Female 87% 5,015,000 aged 65–84, Female 68% 2,203,000 aged ≥85, Female 76% Catheter: Unclear	Hospital (Emergency Department)	Yes	Altered Mental Status ICD - 9 code 780.97; or documentation of disorientation; or presence of reason for visit ICD - 9 codes 5840.0, 5841.0, or 5842.0	Bacteriuria Not Stated UTI ICD-9 CM codes for UTI; or cystitis; or pyelonephritis; (590, 595.0, 595.89, 595.9, or 599.0)	Altered mental status was present in 7% of those aged 65–84, and 13% of those aged ≥85, with UTI. Compared to adults aged 18 to 64 years, those aged ≥85 with UTI were more likely to present with altered mental status. (Adjusted OR = 2.5, 95% CI = 1.3–5.0; *p* = 0.009) Nursing home residents more likely to present with altered mental status (Adjusted OR 4.8 95% CI 2.9–7.8 < 0.001)
*Gau* et al. *2009* [67]	Cross-Sectional, case control retrospective	154 bacteriuric patients Age: ≥65 years, mean 83, SD 8 Female: 84% Catheter: 51% Control group 142 non-bacteriuric patients Age: ≥65 years, mean 82, SD 8 Female: 75% Catheter: 37%	Hospital	Partial	Delirium Defined by delirium, acute confusion, or mental status change as documented on admission	Bacteriuria ≥5 × 10^4^ cfu/ml of a single uropathogen, pyuria, or nitrate positive test results UTI Positive urine culture and atleast local symptoms, fever, delirium (mental status change) or other symptoms (lower abdominal pain, falls, emesis)	46 out of 154 (30%) patients with bacteriuria had delirium Patients with bacteriuria were more likely to have Delirium OR 5.1 (95% CI 2.5–10, *P* < 0.05) 40 out of 104 (39%) patients with UTI had delirium Patients with UTI were more likely to have delirium in comparison to patients with asymptomatic bacteriuria. OR 4.6 (95% CI 1.8–12, p < 0.05)
*Levkoff* et al. *1988* [61]	Retrospective Case Controlled	117 Patients with Delirium Age: ≥60, 54 > 80+ Female: 65% Catheter: Unclear	Hospital	No	Delirium ICD-9 Codes for Delirium	Bacteriuria Not Stated UTI Discharge ICD-9 Codes for UTI	37 out of 117 (32%) with delirium had UTI UTI was associated with delirium OR 3.05 (95% CI 2.01–4.50)
*Lin* et al. *2010* [62]	Cross-sectional; Retrospective	Total 1,968,527 hospitalizations with CHF, UTI, pneumonia or lower limb orthopaedics Age: ≥ 18 years 1,952,301 without delirium Age: ≥ 18 years median age 75 female 60% Catheter: Unclear 16,226 with delirium Age: median age 83, Female 63% Catheter: Unclear	Hospital	Partial	Delirium 6 ICD-9 Codes (drug-induced delirium, presenile dementia with delirium, senile dementia with delirium, vascular dementia with delirium, subacute delirium or delirium not otherwise specified)	Bacteriuria Not Stated UTI CMS-DRG classifications kidney/urinary tract infections (DRGs 320–321)	2700 out of 254,000 (1.1%) patients with UTI presented with delirium on admission 3750 out of 254,000 (1.5%) patients with UTI had delirium at any time during hospitalisation Multivariate models for predicting delirium produced, however UTI used as reference group.
*Lin* et al. *2010* [63]	Cross- Sectional; Retrospective	26,057,988 hospitalizations with CHF, UTI, pneumonia or lower limb orthopaedics Age: ≥ 18 years 25,806,657 without delirium Age: ≥ 18 years median age 74 female 60% Catheter: Unclear 251,331 with any delirium Age: ≥ 18 years median age 83, Female 63% Catheter: Unclear	Hospital	Partial	Delirium 6 ICD-9 Codes (drug-induced delirium, presenile dementia with delirium, senile dementia with delirium, vascular dementia with delirium, subacute delirium or delirium not otherwise specified) Non-dementia, Non-drug Delirium 2 ICD-9 Codes (subacute delirium or delirium not otherwise specified)	Bacteriuria Not Stated UTI CMS-DRG classifications kidney/urinary tract infections (DRGs 320–321)	58,000 out of 3,158,000 (1.8%) patients with UTI had any delirium 38,000 out of 3,158,000 (1%) patients with UTI had non-dementia, non-drug delirium Yearly prevalence of any delirium in patients with UTI 16.6–20.9/1000 Multivariate models for predicting delirium produced, however UTI used as reference group
*Lixouriotis* et al. *2011* [53]	Cross-sectional; Retrospective	9 patients with Delirium Age: mean 76, range 58–83 Female: 44% Catheter: Unclear	General Practice	No	Delirium ICD-10	Bacteriuria Not Stated UTI Not Stated	2 out of 9 (22%) patients with delirium had UTI
*Manepalli* et al *1990* [64]	Cross-Sectional; Retrospective	407 patients Age: Not Stated Female: Not Stated Catheter: Unclear Of the 14 patients with UTI and delirium Age: 81.9 years, range 70–93	Hospital (Psychogeriatric Unit)	Partial	Delirium ICD-9	Bacteriuria Not Stated UTI ICD - 9	14 out of 83 (17%) patients with UTI had delirium 14 out of 54 (26%) patients with delirium had UTI
*Rothberg* et al. *2013* [65]	Cohort; Retrospective	225,028 Age: ≥ 65 years, median 82; Female: 58% Catheter: Unclear	Hospital (Admissions)	No	Delirium Defined as on Day 3 or later prescribed an antipsychotic or placed into restraints	Bacteriuria Not Stated UTI ICD-9-CM	944 out of 20,986 (4.5%) patients with UTI had delirium
*Sabzwari* et al. *2014* [73]	Cross-Sectional; Retrospective	464 patients Age: ≥ 65 years, mean 72.7 SD 6.4; Female: 42% Catheter: Unclear	Hospital (Admission)	Partial	Delirium Key words in clinical notes: acute confusion, acute mental status changes, fluctuating consciousness, acute agitation and organic brain syndrome	Bacteriuria Not Stated UTI Not Stated	17 out of 43 (40%) patients with UTI had delirium 17 out of 101 (17%) patients with delirium had UTI 17% of patients with delirium had UTI compared to 7% of patients without delirium. Adjusted OR 3.1 (95% CI 1.5–6.8, *p* < 0.005)
*Schultz* et al *1991* [57]	Cohort; Prospective	65 residents Age: Not reported Female: Unclear Catheter: Unclear	Nursing Home	Yes	Delirium Not Stated Altered Mental Status Not Stated	Bacteriuria Not Stated UTI significant change in condition + new +ve urine culture (≥10^4^ cfu/ml for gram positive or ≥ 5 × 10^4^ cfu/ml for gram negative organisms) + ≥10 WBCs per high power field.	3.4% of residents with UTI had delirium 12% of residents with UTI had altered mental status
*Silver* et al. *2009* [58]	Cohort; Prospective	335 Patients Age: ≥ 18 years, mean 68, Female: 36% Catheter: 51%	Hospital	No	Confusion or Altered Mental Status Clinical Notes	Bacteriuria > 10^4^ cfu/ml on urine culture Catheter: 10^2^ cfu/mL on urine culture UTI Bacteriuria and either fever without another explanation or ≥ 1 urinary symptom	77 out of 137 (56%) patients with positive urine cultures had confusion or Altered mental status compared to 114 out of 198 (58%) patients with negative urine cultures (*p* = 0.82) 19 out of 34 (56%) UTI patients presented with confusion or altered mental status compared to 44 out of 67 (66%) patients with asymptomatic bacteriura (*p* = 0.17)
*Sundvall 2014* [11]	Cross-sectional; Prospective	421 residents Age (Female): mean 87 years, SD 6.4, range 63–100 Age (Male): mean 85 years, SD 7.1, range 65–100 Female: 70% Catheter: 0%	Nursing Home	Partial	Confusion Nursing Clinical Notes	Bacteriuria ≥10^5^ cfu/ml on urine culture or if signs of possible UTI present: ≥10^3^ for *E. coli* or males with Klebsiella and E.Faecalis; or ≥ 10^4^ women with Klebsiella and E.Faecalis. UTI Not Stated	3 out of 22 (14%) residents with confusion had bacteriuria 3 out of 135 (2.2%) residents with bacteriuria had confusion Residents with bacteriuria were less likely to have confusion OR 0.15 (95% CI 0.033–0.68, *p* = 0.014)
*Sundvall 2011* [69]	Cross-sectional; Prospective	651 residents Age (Female): mean 86 years, SD 7.4, range 46–102 Age (Male): mean 82 years, SD 7.8, range 54–99 Female: 74% Catheter: 0%	Nursing Home	Yes	Confusion Nursing Assessment	Bacteriuria ≥10^5^ cfu/ml on urine culture or if signs of possible UTI present: ≥10^3^ for E. coli or males with Klebsiella and E.Faecalis; or ≥ 10^4^ women with Klebsiella and E.Faecalis. UTI Not Stated	Correlation between bacteriuria with E. Coli and confusion OR 1.8 (95% CI 0.96–3.6, *p* = 0.067) Correlation between bacteriuria and confusion OR 1.9 (95% CI 1.0–3.5, *p* = 0.044)

### Characteristics of included studies

There were four large retrospective cross-sectional studies, and among the remaining studies the number of patients in each study varied considerably from small community samples of 9 to larger hospital samples of 710 (Tables 3 and 4). The majority of the studies identified were cross-sectional in design. Approximately half of the studies had an entirely elderly population ≥ 65 years (*n* = 10), with the other half of studies having populations deemed to be representative of an elderly population by median or mean age ≥ 65 years (n = 10). In the two remaining studies, one conducted in a nursing home and the other in a psychogeriatric unit, the demographics of the patient sample were not provided. They were believed to be representative of an elderly population by their care setting. The proportion of participants with urinary catheters was unclear in the majority of included studies (*n* = 14). In the remaining studies, urinary catheter rates were high, 37–51% (*n* = 3), low 1.8–5.5% (*n* = 2) and none (n = 3). The majority of the studies were conducted in a hospital setting (n = 14), followed by nursing homes (*n* = 6) and community settings (*n* = 2). Interestingly, only two of the included studies had the explicit aim of exploring the association between confusion and UTI; however, ten studies did partially explore this association.

### Results of individual studies

Among the included studies, the rate of confusion in patients with suspected UTI was most commonly reported (*n* = 13) followed by the rate of suspected UTI in patients with confusion (*n* = 10). Some studies reported the rate of confusion in patients with bacteriuria (*n* = 4) and one study reported the rate of bacteriuria in patients with confusion. The majority of studies reported confusion as delirium (*n* = 15), followed by a few reporting confusion (*n* = 3), altered mental state (*n* = 2), and mental status changes (n = 1), with one study reporting both delirium and altered mental status [57]. Twelve studies analysed the correlation between suspected UTI or bacteriuria and confusion (Tables 3 and 4).

### Synthesis of results

No study used validated definitions of both confusion and UTI, so this association could not be reliably established. Only one study by Juthani-Metha et al. had an acceptable definition of confusion and an acceptable definition of bacteriuria. They found an association between bacteriuria and confusion with the relative risk being 1.4 (95% CI 1.0–1.7, *p* = 0.034) [3].

## Discussion

### Summary of the evidence

Following this review, it is evident that all of the studies which have explored the association between suspected UTI and confusion are methodologically flawed, due to poor case definition for UTI or confusion, or inadequate control of confounding factors introducing significant bias. Subsequently, no accurate conclusions about the association between UTI and confusion can be drawn. One study of acceptable quality shows an association between confusion and bacteriuria. However, this sample of patients in whom they tested bacteriuria and pyuria were patients already suspected of having a UTI, introducing a bias into their calculation [3]. In summary, none of the 22 publications had sufficient methodological quality to enable valid conclusions.

### Frailty

Frail residents are more likely to have bacteriuria [74]. Frailty also predisposes for cognitive decline [75, 76]. Hence, there might be an indirect link between confusion and bacteriuria, easily misinterpreted as UTI causing confusion. This might explain some of the trends found in the existing literature.

### Confusion linked to acute cystitis or pyelonephritis

Studies including hospitalised patients are likely to also include patients with pyelonephritis, a condition likely to result in confusion in a fragile elderly person. However, the typical nursing home situation usually involves the suspicion of confusion caused by a lower UTI (acute cystitis) in an afebrile patient.

The primary aim of this review was not to evaluate the association between pyelonephritis and confusion. The primary question was if lower UTI with no fever in residents without a urinary catheter, with or without localised symptoms such as acute dysuria, urgency or frequency, is associated with confusion. This review concludes that current evidence does not provide a clear answer to this question.

### Strengths and limitations of the review

The strengths of this review are mainly due to its methodological quality; that it utilised a broad search strategy, with no limits to age or date applied. This allowed for studies that were representative of an elderly population and without the explicit aim of reporting the relationship between confusion and UTI to be identified. Another strength of this review was the registration of a protocol with pre-specified objectives and methods. The use of a second reviewer independently assessing the quality of selected studies also increases the quality of the review. Limitations included limiting articles to English and being unable to assess the eligibility of the unobtainable full-texts. This review also did not attempt to include studies from the unpublished literature, introducing possible publication bias.

## Conclusion

Insufficient evidence is available to accurately determine if an afebrile lower UTI in elderly patients without an indwelling urinary catheter causes confusion. Although studies exist that suggest there may be an association, they are significantly limited by their methodological quality. This is largely due to the frequent use of unreliable criteria for UTI and confusion, and frequently poor controlling for confounding factors. A reasonable attempt to resolve this issue are the McGeer and Loeb criteria [1, 8, 19]. However, it should be kept in mind that in the case of confusion these criteria are expert recommendations that cannot be confirmed due to the lack of an appropriate gold standard. This review highlights the importance of conducting well-designed studies and demonstrates that further high-quality research exploring the relationship between lower urinary tract infection and acute confusion is required. A well-designed, large observational study with validated criteria for UTI and confusion may provide further insight into this association. However, the optimal solution to clarify this issue would be a randomized controlled trial comparing the effect of antibiotics versus placebo in patients with new onset or worsening confusion and presence of bacteriuria while lacking specific urinary tract symptoms.

## Abbreviation

UTI: Urinary tract infection
